# Advances in Spinal Cord Neuromodulation: The Integration of Neuroengineering, Computational Approaches, and Innovative Conceptual Frameworks

**DOI:** 10.3390/jpm13060993

**Published:** 2023-06-13

**Authors:** Pierre-François Pradat, David Hayon, Sophie Blancho, Pauline Neveu, Mohammed Khamaysa, Nicolas Guerout

**Affiliations:** 1 Laboratoire d’Imagerie Biomédicale, Sorbonne Université, CNRS, INSERM, 75013 Paris, France; mohammed.khamaysa@sorbonne-universite.fr; 2 APHP, Département de Neurologie, Hôpital Pitié-Salpêtrière, Centre Référent SLA, 75013 Paris, France; 3 Northern Ireland Centre for Stratified Medicine, Biomedical Sciences Research Institute Ulster University, C-TRIC, Altnagelvin Hospital, Derry/Londonderry BT47 6SB, UK; 4 Institut Pour la Recherche Sur la Moelle Epiniere et l’Encéphale (IRME), 25 Rue Duranton, 75015 Paris, France; 5 Clinique Saint-Roch, Service d’Anesthésie, 56 Rue de Lille, 59223 Roncq, France; dhayon.cliniquesaintroch@lna-sante.com; 6 Saints Pères Paris Institute for the Neurosciences, Université Paris Cité, CNRS UMR8003, 75006 Paris, Francenicolas.guerout@u-paris.fr (N.G.)

**Keywords:** spinal cord stimulation, epidural stimulation, non-invasive stimulation, transcutaneous electric stimulation, transcutaneous magnetic stimulation

## Abstract

Spinal cord stimulation (SCS) is an approved treatment for intractable pain and has recently emerged as a promising area of research for restoring function after spinal cord lesion. This review will focus on the historical evolution of this transition and the path that remains to be taken for these methods to be rigorously evaluated for application in clinical practice. New developments in SCS are being driven by advances in the understanding of spinal cord lesions at the molecular, cellular, and neuronal levels, as well as the understanding of compensatory mechanisms. Advances in neuroengineering and the computational neurosciences have enabled the development of new conceptual SCS strategies, such as spatiotemporal neuromodulation, which allows spatially selective stimulation at precise time points during anticipated movement. It has also become increasingly clear that these methods are only effective when combined with intensive rehabilitation techniques, such as new task-oriented methods and robotic aids. The emergence of innovative approaches to spinal cord neuromodulation has sparked significant enthusiasm among patients and in the media. Non-invasive methods are perceived to offer improved safety, patient acceptance, and cost-effectiveness. There is an immediate need for well-designed clinical trials involving consumer or advocacy groups to evaluate and compare the effectiveness of various treatment modalities, assess safety considerations, and establish outcome priorities.

## 1. Introduction

Spinal cord (SC) lesions are a major cause of disability globally, affecting both children and adults. These lesions can be caused by various conditions, including traumatic, vascular, tumour, infectious, inflammatory (such as multiple sclerosis), or neurodegenerative (such as motor neuron disease) origin. Among these, SC injuries (SCI) of traumatic origin mainly caused by falls and road accidents in developed countries are culprits [1,2]. Over the past decade, the incidence of cervical SCI due to falls, usually low-energy trauma and associated with pre-existing degenerative changes, in older adults has increased, making it an important public health concern [3,4,5,6].

Spinal cord injury exerts a profound and highly impactful effect on both patients and their families, leading to a high burden. Patients with SCI can experience a range of symptoms, including sensorimotor deficits, respiratory failure, bladder and/or bowel dysfunction, neurogenic and musculoskeletal pain, spasticity, sexual dysfunction, cardiovascular dysautonomia, and psychological distress. Furthermore, patients with SCI are at risk for several common complications, including venous thromboembolism, soft tissue and joint contractures, pressure injuries, orthostatic hypotension, heterotopic ossification, dependent oedema, and osteopenia/osteoporosis.

The burden of the SCI expands also to medical care systems. A recent epidemiological study considered all causes of SCI, based on data from the Global Burden of Disease Study 2019, estimates that there are 0.9 million incident cases and 20.6 million prevalent cases of SCI worldwide [2]. In the United States, medical care costs for these injuries have been estimated to be around $4 billion [7]; and in Canada, a public health study estimated that the net lifetime cost of SCI was $336,000 per person [8].

Improvements in critical care during the acute phase have resulted in an important reduction in mortality within the first two years following injury [9]. Conversely, although there have been advancements in long-term rehabilitation care during the chronic phase, it appears that the improvements in extending the lifespan of individuals with SCI have been overstated [9]. This emphasises the importance of developing new therapeutic approaches specifically targeting chronic SCI patients to enhance long-term functional outcomes and prevent secondary complications. This review aims to provide a brief and critical overview of the development of SCS for SC lesions, highlighting the transition from pain management to the concept that SCS could improve global function [10]. We will focus on the historical evolution of this approach and the path that remains to be taken for these methods to be rigorously evaluated for application in clinical practice.

## 2. The First Era of SCS: The Management of Pain

The origins of electrostimulation are often traced to the first-century AD physician Scribonius Largus, who served in the court of the Roman Emperor Claudius. Largus documented his work, Compositiones Medicamentorum, as a technique for treating gout by placing a live black torpedo fish under the patient’s feet, the patient would stand on a damp seashore, when the pain begins until the entire foot and leg up to the knee becomes numb. This method not only relieved the pain but also prevented new pain from occurring. Largus also advised patients to sit in electrified saltwater pools containing Mediterranean torpedo fish, or to place the black torpedo (Torpedo Nobiliana) on the forehead between the eyebrows and wait for the discharge of the fish to numb the patient’s senses.

The current concept of SCS is based on gate control theory, which states that pain signals transmitted through small fibres can be inhibited by the simultaneous activation of cutaneous touch stimuli mediated through larger myelinated fibres [11]. Following the successful experimental demonstration of the effect of SCS on pain in cats in the late 1960s [12], the minimally invasive procedure of implanting stimulating electrodes in the epidural space has been increasingly used for various conditions associated with intractable pain [13,14,15]. The use of neurostimulators has expanded over time, with an estimated 27,500 devices being implanted in 2007 [16].

## 3. The Second Era: Could SCS Also Improve Function after SC Damage?

### 3.1. Improved Understanding of the Targets of Spinal Cord Neuromodulation

The development of SCS is based on advances in neuroscience that have led to a better understanding of the structural and functional compensatory mechanisms that follow SCI. These compensatory mechanisms occur at neuronal, axonal, spinal, and cerebral network levels, and include changes in neuronal excitability, rostral and caudal axonal collaterals formation at the site of injury, and the reorganisation of cortical maps and subcortical structures [17].

**Figure 1:**
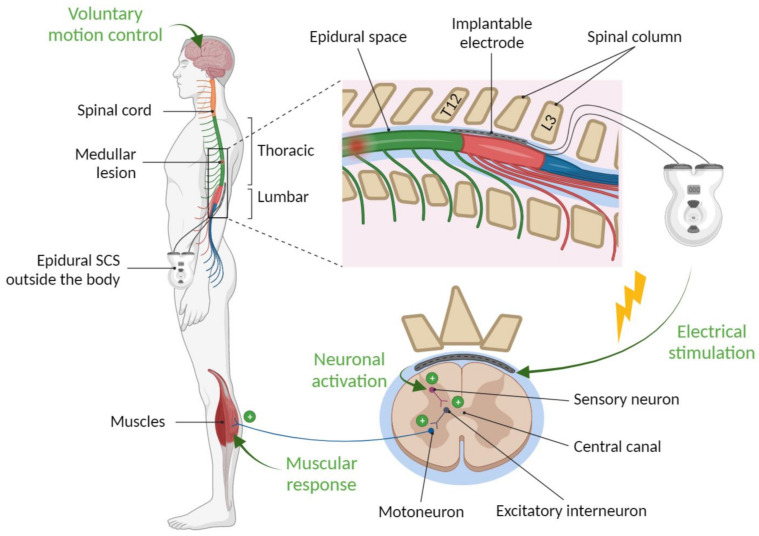
Principle of epidural stimulation: electrode placement and mechanism primarily mediated by the recruitment of sensory fibers in the posterior roots.

Although the underlying mechanisms are not fully understood, it is widely accepted that the neuromodulatory effects of epidural SCS are mediated primarily by the recruitment of sensory fibres in the posterior roots (Figure 1). Activation of proprioceptive afferents causes trans-synaptic modulation of various spinal circuits, such as the spinal motor network (also known as the central pattern generator). Recent research has identified a specific population of excitatory spinal interneurons that mediate the recovery effect of SCS [18]. The effect of SCS has also been shown to be related to the reorganisation of supraspinal circuits that are dependent on the mesencephalic locomotor region [19].

There is limited research on the effects of SCS in the acute or subacute phase of SCI. Although this area is critical for the potential application of SCS on patients with the early stages of SCI. Encouraging studies in rodents have shown positive effects on several signalling pathways involved in the pathogenesis of SCI. SCS has shown beneficial effects on neuron and oligodendrocyte survival, the preservation of myelin, and the modulation of astrocytes, microglia, and macrophage activation [20,21].

### 3.2. From Neuroscience to Clinical Efficacy

There is an empirical observation going back approximately fifty years, suggesting that functional improvement might occur after epidural stimulation [22]. After performing this intervention to treat refractory pain in a young person with multiple sclerosis (MS), clinicians observed unexpected improvement in spasticity, gait, and fatigue. In the following years, after a period of skepticism within the medical community, a publication in the Lancet also reported an improvement in motor, sensory, and urinary functions in two MS patients treated with epidural stimulation [23]. This work opened new perspectives for evaluating the effect of epidural stimulation on residual neurological deficits in MS. According to a recent systematic review, while the individual studies had a low level of evidence, there is evidence to suggest that SCS is effective in improving neurological function in patients with MS [24]. The authors analysed seven trials involving 373 MS patients treated with SCS. Overall, sustained improvement was observed in motor dysfunction (56%), sphincter dysfunction (67%), and neuropathic pain (82%). Since the 2000s, SCS has been introduced to the field of SCI and has become an area of great interest for clinical trials in the SCI field. A seminal publication reported the effects of epidural stimulation on a 23-year-old patient with paraplegia following a road accident [25]. Improvements were observed in standing with a load, walking, and voluntary leg movements in the supine position. Recently, a systematic literature review identified 40 eligible articles involving 184 patients with incomplete or complete SCI treated with epidural stimulation [26]. Most studies reported improvements in the outcome measures assessed, whether motor function or dysautonomia.

Recent developments in SCI have brought about a shift, with the introduction of new approaches based on spatiotemporal neuromodulation [27], which aims to improve locomotion by stimulating appropriate muscles based on their spatial location in the SC (e.g., flexor vs. extensor, hip vs. ankle, etc.) in accordance with the rhythmicity of balance position without negatively affecting endogenous proprioceptive information [28]. By using an implanted pulse generator with real-time trigger functions, it is possible to deliver spatially selective stimulation trains in the lumbosacral SC with timing consistent with expected movement [27]. After a few months, participants regained voluntary control of previously paralysed muscles without stimulation and were able to walk or cycle in an ecological environment during spatiotemporal stimulation. Recently, this approach has been optimised with a new set of electrodes targeting all dorsal roots, computationally based electrode positioning, and software that supports the rapid configuration of activity-specific stimulation programs that reproduce the natural activation of underlying motor neurons for each activity [29]. In a single day, the activity-specific stimulation programs enabled these three individuals to stand, walk, cycle, swim, and control trunk movements. A new milestone was reached by demonstrating that cervical SCS can be applied to brain pathologies, resulting in a significant effect on orthostatic hypotension in multiple system atrophy [30] or upper limb deficits after stroke [31].

## 4. The Development of Non-Invasive Approaches

Spinal cord stimulation has expanded to include less invasive modalities where stimulation is delivered through the skin surface. The first publications date from 1996 and 2009, respectively [32,33]. Interest in these modalities is growing, as evidenced by the recent increase in publications [34]. The authors speculate that the increasing popularity of transcutaneous SCS is not only due to the fact that it is non-invasive and accepted by patients, but also due to the lower costs of research and development, potentially shorter routes to market, and easier recruitment of participants [34]. Transcutaneous (or percutaneous or trans-spinal) stimulation (TESCS), like epidural SCS, was first used to relieve chronic pain in patients and later to improve motor function and spasticity after focal SCI [35,36]. The mechanisms are thought to be the same as those underlying the effects described for epidural SCS, namely activation of sensory afferents [37,38]. Another non-invasive stimulation technique under development is repetitive trans-spinal magnetic stimulation (rTSMS). This technique uses magnetic fields to stimulate specific SC areas and has been shown to promote functional recovery in different rodent models of acute SCI. The effects of rTSMS at the cellular and molecular levels include an increase in the survival of neurons and oligodendrocytes, the preservation of myelin, and the proliferation of endogenous ependymal stem cells [39,40,41]. Moreover, rTSMS can induce axonal regrowth in the injured SC [41].

## 5. Time for Improving Clinical Trials Designs

Although there is a growing body of evidence supporting the effectiveness of SCS in improving function in patients with chronic SCI, and even though, it is a minimally invasive and safe procedure that has been tested for decades, meta-analyses [26,42] have concluded that current studies cannot definitively determine the global efficacy of SCS and which patients benefit most from this intervention. A scoping review of SCS research on the recovery of motor, sensory, and autonomic functions (Table 1) [34] has recently highlighted the main limitations in existing studies. There is an urgent need to establish common guidelines for the design and conduct of clinical trials evaluating SCS procedures.

It is crucial to integrate SCS into the overall care of patients, especially with intensive and task-specific rehabilitation, which is a critical factor to consider in the design and interpretation of clinical trials. Although the mechanisms of rehabilitation are multifactorial, they share with SCS the ability to be primarily afferent-driven by promoting proprioceptive input to the spinal cord [43,44]. Task-specific rehabilitation has been shown to be more beneficial than conventional rehabilitation for motor recovery in people with chronic SCI [45,46]. This is because intensive practise of actions or functional tasks relies on the fact that motor performance can be shaped and retrained in response to specific sensory input [47]. A recent report has shown that a combination of rTSMS and task-specific rehabilitation can improve trunk and sitting functions in patients with chronic quadriplegia [48]. The recent extensive development of new robotic rehabilitation tools, such as exoskeletons that activate task-dependent proprioceptive inputs, offers promising prospects for facilitating and maintaining the long-term functional effects of SCS.

## 6. Perspectives and Conclusions

Neuromodulation therapies involving external and implantable devices are being increasingly utilised in clinical practice for treating a range of neurological and psychiatric conditions. This scoping review highlights how these therapies have now entered a new era with regard to restoring function following spinal lesions. Advances in the neurosciences, neuroengineering, and the computational sciences have led to the development of new conceptual frameworks in this area. It is important to highlight that SCS approaches need to be integrated into comprehensive care, particularly in conjunction with intensive and task-specific rehabilitation. The pioneering work of neuroscientists, such as Hebbian theory [49], reminds us of the “use it or lose it” concept, whereby inactivity leads to the pruning of neural reorganization, and a return to activity strengthens synaptic connections. The neuromodulation approach is integrated within the ongoing advancements of other therapeutic strategies that aim to facilitate spinal network recovery after SCI. These include neuropharmacological methods and the transplantation of stem cells such as embryonic serotoninergic cells or differentiated cells such as Schwann cells or olfactory ensheathing cells. Combining these approaches in the future could yield significant benefits. SCS has received considerable media attention, and there is hope that it will benefit patients. However, for these exciting approaches to be translated into practice and benefit a large number of patients, considerable efforts will be needed from the medical community to build international collaborations, evaluate SCS through structured clinical trials, and engage with consumers and advocacy groups to identify treatment and outcome priorities.

## Figures and Tables

**Table 1:** Limitations in the design and report of the existing trials assessing the effect of SCS on function in SCI patients.

Shortcomings	Avenues
Disease Heterogeneity	Subgroup analysis based on lesion level and post-traumatic delay
Small sample size	Multicenter trial to increase sample size
Incomplete description of the therapeutic procedures	Need to document the device used and the stimulation parameters Use common technical terminologies Detail the timing of the stimulation, follow-up, rehabilitation activities, and sample sizes.
Incomplete clinical outcomes focusing on lower motor recovery and ambulation	Consider sexual, autonomic, bladder, and bowel function Quality of life scales.
Poorly reported risk and safety issues	Document adverse events Inclusion of a designated and independent data monitoring and/or safety committee
Absence of objective surrogates	Include biomarkers (MRI, electrophysiology)
Lack of comparative studies	Need for comparative studies of the 3 forms of SCS: epidural SCS, electric and magnetic transcutaneous SCS.
Absence of patient participation	Engagement and participation of consumers or advocacy groups to identify treatment and outcome priorities Need for patient’s reported outcomes
Dissemination	Results of any clinical trial should comply with standardised checklist of the CONSORT guidelines Avoid publication bias in favor of the positive studies and publish negative studies

Abbreviation: SCI (Spinal Cord Injury), SCS (spinal cord stimulation).

## Data Availability

Not applicable.
